# Smart Sensors for Structural Health Monitoring and Nondestructive Evaluation

**DOI:** 10.3390/s24020603

**Published:** 2024-01-17

**Authors:** Zenghua Liu

**Affiliations:** Faculty of Information Technology, Beijing University of Technology, Beijing 100124, China; liuzenghua@bjut.edu.cn

## 1. Introduction

During manufacturing, processing, and usage, various types of damage may be caused in structures. For example, corrosion and fatigue cracks are common defects in metal plates, while the main defects in composite plates are delamination, debonding, etc. Thus, it is important to develop defect detection and monitoring techniques to ensure the integrity of structures [1,2,3]. Structural health monitoring (SHM) and nondestructive evaluation (NDE) technologies can be used to identify defects or damages and evaluate the health status of components and systems to avoid structural failure or catastrophes. NDE is usually a technique for detecting offline defects without destroying structures using removable sensors. In contrast, SHM is a potential alternative whereby the global and local security state of a structure is permanently monitored through embedded or attached sensors or sensor arrays. By monitoring the condition, or change in conditions, of a structure, the detection of damage at any location within a structure becomes possible [4,5,6]. SHM and NDE both aim to assess the integrity of a structure nondestructively and these fields often overlap, whether that be to a greater or lesser extent [7].

With complex working conditions of structures, including harsh and high-temperature environments, increasing structure scale, and increasing detection requirements, SHM and NDE are progressively becoming more challenging to implement [8,9,10,11]. A lot of techniques with different working mechanisms, such as the piezoelectric effect [12], magnetostriction effect [13], and magnetic effect [14], can be used for SHM and NDE. In order to detect how safe a structure is, it is crucial to use appropriate sensors and sensor arrays to obtain effective information to try to evaluate a safety status using various signal processing methods. A major development in the fields of SHM and NDE over the past few decades has been the development and use of smart sensors.

In short, the development and application of sensors are key research topics in the areas of SHM and NDE. This Special Issue “Smart Sensors for Structural Health Monitoring and Nondestructive Evaluation” has collected the most recent original contributions relating to all facets of smart sensors utilized in structural health monitoring and nondestructive evaluation. The call for papers for this Special Issue included topics such as sensors and sensor arrays, sensor modeling and simulation, SHM systems and technology, nondestructive testing and evaluation, structural diagnosis and performance evaluation, signal processing, artificial intelligence applications in SHM and NDE, system and instrument development, and field applications of SHM and NDE.

## 2. Overview of Published Papers

In this context, this Special Issue includes 33 papers focused on the latest advancements in the field of smart sensors for structural health monitoring and nondestructive evaluation. Each of the 33 original contributions (2 review papers and 31 research papers) accepted for publication have undergone a rigorous review process by a minimum of two expert reviewers across at least two rounds of revision. The papers published in the current Special Issue are briefly summarized as follows:

In contribution 1, the authors present an overview of recent progress in the field of piezoelectric materials and sensors for structural health monitoring. The article commences with a brief introduction to the fundamental physical science of the piezoelectric effect. Emphases are placed on piezoelectric materials engineered using various strategies and applications of piezoelectric sensors for structural health monitoring. Finally, challenges, along with opportunities for future research and the development of high-performance piezoelectric materials and sensors for structural health monitoring, are highlighted.

In contribution 2, the authors present a systematic literature review on the state-of-the-art electrochemical methods and physical methods used so far for corrosion monitoring that are compatible with low-cost sensors and data acquisition devices for metallic and concrete structures. In addition, special attention is paid to the use of these devices for corrosion monitoring and detection for in situ applications in different industries. This analysis demonstrates the possible applications of low-cost sensors in the corrosion monitoring sector. In addition, this study provides scholars with preferred techniques and the most common microcontrollers to overcome corrosion monitoring difficulties in the construction industry.

In contribution 3, the authors propose a two-step physics- and machine learning (ML)-based electromechanical impedance (EMI) measurement data evaluation approach for sandwich face-layer debonding detection and size estimation in SHM applications. This approach was shown to be robust against unknown artificial disturbances and it outperformed a previous method for debonding size estimation.

In contribution 4, the authors propose a novel baseline-free method for damage localization using Lamb waves based on a hyperbolic algorithm. This method employs a special array with a relatively small number of transducers and only one branch of the hyperbola. The novel symmetrical array was arranged on plate structures to eliminate the direct waves. The imaging results showed that both the damages outside and inside the diamond-shaped arrays could be localized, and the positioning error reached its maximum for the diamond-shaped array using the minimum size.

In contribution 5, the authors discuss the need for surface monitoring of municipal solid waste (MSW) landfills. A properly 3D-mapped landfill mass was the basis for ensuring the geotechnical safety of the restored landfill. Based on archival data and current measurements of Radiowo landfill (Poland), this study compared the advantages and limitations of the following measurement techniques: linear and angular measurements, satellite measurements, TLS, and UAV scanning and photogrammetry, considering specific conditions regarding the location and vegetation of the landfill. Solutions for long-term monitoring were proposed, considering the cost and time resolution necessary for creating a differential model of landfill geometry changes.

In contribution 6, the authors present a novel adaptive filtering approach to enhance the signal-to-noise ratio (SNR) of a measured ultrasonic signal from the inspection of a stainless steel component, enabling the detection of hidden flaws under strong noise. The performance of the proposed method for SNR enhancement was evaluated by both the simulated and experimental signal and its effectiveness was successfully demonstrated.

In contribution 7, the authors propose a method to simultaneously estimate the mechanical parameters of vehicles, bridges, and road unevenness with only a few constraints. Signals from acceleration sensors attached to vehicles traveling on bridges were processed. Methods were proposed to estimate the modal parameters of bridges and road unevenness from vehicle vibrations on a case-by-case basis. The road surface irregularities estimated by the proposed method were compared with the measured values, which were somewhat smaller than the measured values.

In contribution 8, the authors propose a deep neural network to detect failures and investigate sensor sensitivity to damage classification for an orbiting satellite hosting a distributed network of accelerometers on a large mesh reflector antenna. The proposed deep learning framework and sensor configuration were shown to accurately detect failures in the most critical area or the structure while opening new investigation possibilities regarding geometrical properties and sensor distribution.

In contribution 9, the authors investigate the effect of the cycle period of a stimulation wave on amplitude-phase results by performing various numeric simulations and laboratory tests. The recommended cycle period from the prediction surfaces was experimentally validated using two samples. After various laboratory experiments, the temperature, amplitude, and phase results validated the previous equations and prediction surfaces relating to the poly(methyl methacrylate) (PMMA) and carbon-fiber-reinforced polymer (CFRP) sample.

In contribution 10, the authors develop a wireless impedance monitoring system to have cheap, mobile, and handy practical features compared to wired commercial impedance analyzers. A Raspberry Pi platform sensor node was designed to acquire impedance signals using a PZT interface technique. The software scheme was designed to operate the Raspberry Pi platform and impedance sensor node. The calibration procedure was designed for the impedance sensor node. The feasibility of the proposed Raspberry Pi platform SSeL-Pi system was experimentally evaluated for PZT interfaces that were subjected to various compressive loadings.

In contribution 11, the authors design a sandwich piezoelectric ceramic transducer and analyze the vibration of each part of the transducer, making use of elastic mechanics and piezoelectric theory. The vibration characteristics of the transducer under different parameters such as voltage and frequency were analyzed, and the accuracy of the vibration model was verified. The results showed that the equivalent simplified model established in this study could effectively be used to design the inherent frequency of the transducer, and the operation of the first-order inherent frequency met the one-dimensional assumptions of this study.

In contribution 12, the authors present research on the performance of composite and monolithic sensors for distributed fiber optic sensing (DFOS). The performance of each DFOS nondestructive tool was investigated in the close vicinity of the cracks—both the new ones, opening within the tension zone, and the existing ones, closing within the compression zone. Qualitative (detection) and quantitative (width estimation) crack analyses were performed and discussed. Finally, examples of actual applications within concrete structures, including bridges, were presented, with some examples of in situ results.

In contribution 13, the authors develop a laser ultrasonic system to indirectly evaluate the preload force of different-frequency piezoelectric bolts and achieve the goal of non-contact excitation and synchronous reception of laser-induced ultrasonic signals using a combination of a smart piezoelectric sensor and a magnetically mounted transducer connector. The results indicate that the proposed system based on a surface-mounted piezoelectric sensor is a promising system for evaluating axial preload change in a connector and fastener and is an additional potential laser ultrasonic system for nondestructive tests.

In contribution 14, the authors propose a new configuration of angled shear vertical (SV) wave EMAT with horizontal magnetization to reduce the influence of a head wave. The results from simulations and experiments showed that the proposed EMAT had a larger signal amplitude and significantly reduced interference in large-incidence angle scenarios. Moreover, it was found that an incidence angle of an SV wave of up to 60 degrees could be achieved, which will help to improve the performance and capability of nondestructive testing.

In contribution 15, the authors present an experimental field study using an unattended corrosion sensor developed on the basis of ultrasound technology to assess the thickness loss caused by general atmospheric corrosion on land close to the sea (coastal regions). The system described here used FPGA, a low-power microcontroller, analog front-end devices in the sensor node, and a Beaglebone black wireless board to post data to a server. Over the course of 5 months, the proposed experiment continuously monitored the corrosion rate in an equivalent corrosion process, showing an average thickness loss rate of 0.134 mm/year.

In contribution 16, the authors propose an improved RAPID (reconstruction algorithm for probabilistic inspection of defect) imaging method based on machine learning (ML) to precisely visualize the location and features of defects in a composite plate. The simulation results showed that the proposed method can intuitively characterize the location and related feature information of a defect and effectively improve the accuracy of defect imaging.

In contribution 17, the authors investigate directional magnetic incremental permeability in an iron–cobalt magnetic sheet. The study revealed that an angle of π/2 between DC (Hsurf DC) and AC (Hsurf AC) magnetic excitations with a flux density Ba at Hsurf DC = 10 kA·m^−1^ constituted the ideal experimental situation and the highest correlated parameter in homogeneous imposed tensile stress. Magnetic incremental permeability was linked to the magnetic domain wall bulging magnetization mechanism; this study thus provided insights for understanding such a mechanism.

In contribution 18, the authors present a measurement system involving the application of 3D cameras for a wind tunnel test. A specific application of displacement measurement using a 3D shape was shown and an analysis of acquired data allowed the authors to obtain the displacement of each side of each structure, for different sections. This proposed system can be seen as a complete system for measuring displacement, with higher accuracy than relying on the uncertainty of 3D sensors, and a system for measuring the 3D geometry of structures in harsh environments.

In contribution 19, the authors propose an efficient semi-analytical method capable of modeling the propagation of flexural waves on cracked plate structures with any form of excitation, based on the same group of vibration characteristics and validated by a non-contact scanning Laser Doppler Vibrometer (LDV) system. The proposed modeling method was based on the superposition of the vibrational normal modes of the detected structure, which could be applied to analyze long-time and full-field transient wave propagations. The experimental results indicated that the proposed semi-analytical method can model flexural waves, and in doing so, crack information can be revealed.

In contribution 20, the authors present the effect of different curing methods on gas permeability with the help of laboratory and on-site tests, showing that inadequate curing leads to increased permeability in the near-surface area of concrete. The measurement results of concrete samples and components with the same composition but varying curing treatments were compared and evaluated. Influences on the quality of concrete, such as concrete composition and environmental factors, were observed.

In contribution 21, the authors deduce the theoretical process of calculating the propagation characteristics of Lamb waves in functionally graded material (FGM) sandwich plates by combining the FGM volume fraction curve and Legendre polynomial series expansion method. For comparison purposes, the Lamb wave dispersion curve of the sliced layer model for the FGM sandwich plate was obtained using the global matrix method. Meanwhile, the FGM sandwich plate was subjected to finite element simulation, which was also based on the layered-plate model. The acoustic characteristic detection experiment was performed via simulation using a defocusing measurement. Thus, the Lamb wave dispersion curves were obtained through V(f, z) analysis.

In contribution 22, the authors thoroughly investigate pulse modulation eddy current (PMEC) for the imaging and assessment of ILC through theoretical simulations and experiments. A semi-analytical model of PMEC evaluation of ILC occurring at the interlayer of two conductor layers was established based on extended truncated region eigenfunction expansion (ETREE) along with an efficient algorithm for the numerical computation of eigenvalues for reflection coefficients of the stratified conductor under inspection. The theoretical and experimental results revealed the feasibility of PMEC for the imaging and evaluation of ILC in stratified conductors.

In contribution 23, the authors investigate the validation of a numerical method from the literature which was used to simulate an H-N source on a complex plate. The location results of the numerical delta-t map technique were compared with those of traditional TOA techniques and an experimental delta-t map technique. The viability of using the FE method was demonstrated to decrease the time and labor required for manually collecting and processing training data whilst maintaining a reasonable degree of source location accuracy with an average error of 3.88 mm. With such high source location accuracy, the specific area of concern requiring inspection using other NDE techniques can be greatly reduced.

In contribution 24, the authors propose an adaptive signal truncation method based on signal difference analysis to improve the defect location accuracy of the probabilistic elliptic imaging algorithm. The experimental results showed that the probabilistic ellipse imaging algorithm, based on the new adaptive signal truncation method, could effectively locate a single defect on a pressure vessel.

In contribution 25, the authors derive an analytical expression for the response of piezoelectric transducers under the action of stress waves to obtain an overall mathematical model of an acoustic emission signal from generation to reception. By comparing the finite element simulations, experimental validation, and analytical modeling, it was found that they were almost consistent in the time and frequency domains, and the presence of the Lamb wave S0 mode was clearly observed. All of this validated the accuracy of the analytical modeling predictions.

In contribution 26, the authors propose a novel time reversal (TR)-based localization method for pipeline leakage. In the proposed method, so-called TR self-adaptive cancellation was developed to improve leak localization resolution. The experimental results showed that the leak positions could be accurately revealed by using the proposed approach. Furthermore, the resolution of the proposed approach was shown to be ten times that of the conventional TR localization method.

In contribution 27, the authors measure the acoustic parameters of guided wave propagation in different cement tailing ratios and different curing times of cemented paste backfills (CPBs). Combined with the uniaxial compression strength of CPB, the relationships between CPB strength and the guided wave parameters were established. Based on the relationship curves between CPB strength and guided wave velocity and attenuation, the guided wave technique was proven to be feasible in monitoring the strength development of CPB.

In contribution 28, the authors propose a method using only two piezoelectric transducers and based on orthogonal matching pursuit (OMP) decomposition to detect damage with the fewest sensors and high resolution. The experimental results showed that the OMP-based algorithm was beneficial for resolution improvement and transducer usage reduction.

In contribution 29, the authors propose a new method for identifying many different types of rail defects. Segment principal component analysis (S-PCA) was utilized to extract characteristics from signals collected by sensors located at different positions. The Support Vector Machine (SVM) model was used to identify different defects depending on the features extracted. The results showed that the defect classification accuracy rates were 96.29% and 96.15% for combining multiple signals, such as the method of single-point excitation and multi-point reception or the method of multi-point excitation and reception at a single point.

In contribution 30, the authors propose a new concept of nonlinearity parameter grouping with multi-frequency excitation as an early failure parameter. An analytical solution of the one-dimensional wave equation was derived with four fundamental frequencies and a total of 64 individual and 30 group nonlinearity parameters. Experimental validation of the approach was conducted on metal plates with different types of cracks and on turbine blades where cracks originated under service conditions. The results showed that the use of multi-frequency excitation offered advantages in detecting cracks.

In contribution 31, the authors present a metamaterial lens design and its use in far-field microwave imaging for subwavelength defect detection in nondestructive evaluation (NDE). Theoretical formulation and numerical studies of the metamaterial lens design were presented, followed by experimental demonstration and characterization of metamaterial behavior. A microwave homodyne receiver-based system was used in conjunction with the metamaterial lens to develop a far-field microwave NDE sensor system. The system was shown to be sensitive to a defect of size 0.17λ × 0.06λ in a Teflon sample. Consecutive positions of the defect with a separation of 0.23λ were resolvable using the proposed system.

In contribution 32, the authors present a coherent optical fiber sensor with adequate sensitivity for detecting acoustic emission (AE) during the propagation of a crack in a ferrous material. The proposed fiber optic sensor was successfully compared, in terms of the SNR (signal-to-noise ratio) and detectable AE energy levels, to commercially available AE piezo-transducers and was proven to be an effective and advantageous alternative for sensing and monitoring fatigue damage in structural applications.

In contribution 33, the authors propose a new triangle eddy current sensor array to increase the level of quantifying hole-edge crack parameters, especially the crack angle. This new sensor array consists of triangular coils instead of planar rectangular coils. The configuration of the novel sensor array, including the excitation current directions and the excitation winding shape, was optimized through simulation. The ability of the proposed sensing film to identify the crack parameters was verified using finite element simulations and experiments. The results showed that triangular coils with the same current directions in circumferentially adjacent coils and opposite current directions in axially adjacent coils achieved better performance in terms of sensor linearity and resolution compared to rectangular coils.

## 3. Conclusions

In this Special Issue, we selected 33 papers that address different topics related to smart sensors for structural health monitoring and nondestructive evaluation in order to delineate the state of the art and the future of this field. We hope that the selected papers may provide useful insights into the research areas of smart sensors for structural health monitoring and nondestructive evaluation, inspiring future work in these rapidly developing research fields.

We would like to thank all of the authors who contributed their work to this Special Issue, as well as all of the reviewers of the submitted papers who dedicated their time and expertise to providing high-quality suggestions and comments that allowed us to finalize a successful Special Issue.

## List of Contribution

1. Ju, M.; Dou, Z.; Li, J.-W.; Qiu, X.; Shen, B.; Zhang, D.; Yao, F.-Z.; Gong, W.; Wang, K. Piezoelectric Materials and Sensors for Structural Health Monitoring: Fundamental Aspects, Current Status, and Future Perspectives. *Sensors* **2023**, *23*, 543. https://doi.org/10.3390/s23010543.
2. Komary, M.; Komarizadehasl, S.; Tošić, N.; Segura, I.; Lozano-Galant, J.A.; Turmo, J. Low-Cost Technologies Used in Corrosion Monitoring. *Sensors* **2023**, *23*, 1309. https://doi.org/10.3390/s23031309.
3. Kralovec, C.; Lehner, B.; Kirchmayr, M.; Schagerl, M. Sandwich Face Layer Debonding Detection and Size Estimation by Machine-Learning-Based Evaluation of Electromechanical Impedance Measurements. *Sensors* **2023**, *23*, 2910. https://doi.org/10.3390/s23062910.
4. Xu, J.; Zhu, W.; Qiu, X.; Xiang, Y. A Novel Baseline-Free Method for Damage Localization Using Guided Waves Based on Hyperbola Imaging Algorithm. *Sensors* **2023**, *23*, 2050. https://doi.org/10.3390/s23042050.
5. Pasternak, G.; Zaczek-Peplinska, J.; Pasternak, K.; Jóźwiak, J.; Pasik, M.; Koda, E.; Vaverková, M.D. Surface Monitoring of an MSW Landfill Based on Linear and Angular Measurements, TLS, and LIDAR UAV. *Sensors* **2023**, *23*, 1847. https://doi.org/10.3390/s23041847.
6. Wu, B.; Yang, H.; Huang, Y.; Zhou, W.; Liu, X. A Novel Adaptive Time-Frequency Filtering Approach to Enhance the Ultrasonic Inspection of Stainless Steel Structures. *Sensors* **2023**, *23*, 1030. https://doi.org/10.3390/s23021030.
7. Shin, R.; Okada, Y.; Yamamoto, K. Discussion on a Vehicle–Bridge Interaction System Identification in a Field Test. *Sensors* **2023**, *23*, 539. https://doi.org/10.3390/s23010539.
8. Angeletti, F.; Iannelli, P.; Gasbarri, P.; Panella, M.; Rosato, A. A Study on Structural Health Monitoring of a Large Space Antenna via Distributed Sensors and Deep Learning. *Sensors* **2023**, *23*, 368. https://doi.org/10.3390/s23010368.
9. Ramos Silva, A.; Vaz, M.; Leite, S.; Mendes, J. Lock-In Thermal Test Simulation, Influence, and Optimum Cycle Period for Infrared Thermal Testing in Non-Destructive Testing. *Sensors* **2023**, *23*, 325. https://doi.org/10.3390/s23010325.
10. Pham, Q.-Q.; Ta, Q.-B.; Park, J.-H.; Kim, J.-T. Raspberry Pi Platform Wireless Sensor Node for Low-Frequency Impedance Responses of PZT Interface. *Sensors* **2022**, *22*, 9592. https://doi.org/10.3390/s22249592.
11. Lu, Y.; Xu, C.; Pan, Q.; Yu, Q.; Xiao, D. Research on Inherent Frequency and Vibration Characteristics of Sandwich Piezoelectric Ceramic Transducer. *Sensors* **2022**, *22*, 9431. https://doi.org/10.3390/s22239431.
12. Bednarski, Ł.; Sieńko, R.; Howiacki, T.; Zuziak, K. The Smart Nervous System for Cracked Concrete Structures: Theory, Design, Research, and Field Proof of Monolithic DFOS-Based Sensors. *Sensors* **2022**, *22*, 8713. https://doi.org/10.3390/s22228713.
13. Ren, G.; Zhan, H.; Liu, Z.; Jiang, W.; Li, R.; Liu, S. Evaluation of Axial Preload in Different-Frequency Smart Bolts by Laser Ultrasound. *Sensors* **2022**, *22*, 8665. https://doi.org/10.3390/s22228665.
14. Qu, Z.; Li, Z.; Yang, R.; Hu, S.; Wang, S. Extending the Incidence Angle of Shear Vertical Wave Electromagnetic Acoustic Transducer with Horizontal Magnetization. *Sensors* **2022**, *22*, 8589. https://doi.org/10.3390/s22228589.
15. Thibbotuwa, U.C.; Cortés, A.; Irizar, A. Small Ultrasound-Based Corrosion Sensor for Intraday Corrosion Rate Estimation. *Sensors* **2022**, *22*, 8451. https://doi.org/10.3390/s22218451.
16. Deng, F.; Zhang, X.; Yu, N.; Zhao, L. An Improved RAPID Imaging Method of Defects in Composite Plate Based on Feature Identification by Machine Learning. *Sensors* **2022**, *22*, 8413. https://doi.org/10.3390/s22218413.
17. Toutsop, B.; Ducharne, B.; Lallart, M.; Morel, L.; Tsafack, P. Characterization of Tensile Stress-Dependent Directional Magnetic Incremental Permeability in Iron-Cobalt Magnetic Sheet: Towards Internal Stress Estimation through Non-Destructive Testing. *Sensors* **2022**, *22*, 6296. https://doi.org/10.3390/s22166296.
18. Marchisotti, D.; Schito, P.; Zappa, E. 3D Measurement of Large Deformations on a Tensile Structure during Wind Tunnel Tests Using Microsoft Kinect V2. *Sensors* **2022**, *22*, 6149. https://doi.org/10.3390/s22166149.
19. Wang, D.-F.; Chuang, K.-C.; Liu, J.-J.; Liao, C.-Y. Modeling Full-Field Transient Flexural Waves on Damaged Plates with Arbitrary Excitations Using Temporal Vibration Characteristics. *Sensors* **2022**, *22*, 5958. https://doi.org/10.3390/s22165958.
20. Ptacek, L.; Strauss, A.; Bos, C.; Peyerl, M.; Torrent, R. Concrete Curing Performance Assessment Based on Gas Permeability Testing in the Lab and on Site. *Sensors* **2022**, *22*, 4672. https://doi.org/10.3390/s22134672.
21. Gao, J.; Zhang, J.; Lyu, Y.; Song, G.; He, C. Lamb Waves Propagation Characteristics in Functionally Graded Sandwich Plates. *Sensors* **2022**, *22*, 4052. https://doi.org/10.3390/s22114052.
22. Liu, Z.; Li, Y.; Ren, S.; Ren, Y.; Abidin, I.M.Z.; Chen, Z. Pulse-Modulation Eddy Current Evaluation of Interlaminar Corrosion in Stratified Conductors: Semi-Analytical Modeling and Experiments. *Sensors* **2022**, *22*, 3458. https://doi.org/10.3390/s22093458.
23. Yang, H.; Wang, B.; Grigg, S.; Zhu, L.; Liu, D.; Marks, R. Acoustic Emission Source Location Using Finite Element Generated Delta-T Mapping. *Sensors* **2022**, *22*, 2493. https://doi.org/10.3390/s22072493.
24. Li, Q.; Luo, Z.; Hu, G.; Zhou, S. A New Probabilistic Ellipse Imaging Method Based on Adaptive Signal Truncation for Ultrasonic Guided Wave Defect Localization on Pressure Vessels. *Sensors* **2022**, *22*, 1540. https://doi.org/10.3390/s22041540.
25. Mu, W.; Gao, Y.; Wang, Y.; Liu, G.; Hu, H. Modeling and Analysis of Acoustic Emission Generated by Fatigue Cracking. *Sensors* **2022**, *22*, 1208. https://doi.org/10.3390/s22031208.
26. Mo, Y.; Bi, L. TR Self-Adaptive Cancellation Based Pipeline Leakage Localization Method Using Piezoceramic Transducers. *Sensors* **2022**, *22*, 696. https://doi.org/10.3390/s22020696.
27. He, W.; Zheng, C.; Li, S.; Shi, W.; Zhao, K. Strength Development Monitoring of Cemented Paste Backfill Using Guided Waves. *Sensors* **2021**, *21*, 8499. https://doi.org/10.3390/s21248499.
28. Mu, W.; Gao, Y.; Liu, G. Ultrasound Defect Localization in Shell Structures with Lamb Waves Using Spare Sensor Array and Orthogonal Matching Pursuit Decomposition. *Sensors* **2021**, *21*, 8127. https://doi.org/10.3390/s21238127.
29. Deng, F.; Li, S.-Q.; Zhang, X.-R.; Zhao, L.; Huang, J.-B.; Zhou, C. An Intelligence Method for Recognizing Multiple Defects in Rail. *Sensors* **2021**, *21*, 8108. https://doi.org/10.3390/s21238108.
30. Mevissen, F.; Meo, M. Nonlinear Ultrasound Crack Detection with Multi-Frequency Excitation—A Comparison. *Sensors* **2021**, *21*, 5368. https://doi.org/10.3390/s21165368.
31. Datta, S.; Mukherjee, S.; Shi, X.; Haq, M.; Deng, Y.; Udpa, L.; Rothwell, E. Negative Index Metamaterial Lens for Subwavelength Microwave Detection. *Sensors* **2021**, *21*, 4782. https://doi.org/10.3390/s21144782.
32. Di Luch, I.; Ferrario, M.; Fumagalli, D.; Carboni, M.; Martinelli, M. Coherent Fiber-Optic Sensor for Ultra-Acoustic Crack Emissions. *Sensors* **2021**, *21*, 4674. https://doi.org/10.3390/s21144674.
33. Fan, S.; Yi, J.; Sun, H.; Yun, F. Quantifying Hole-Edge Crack of Bolt Joints by Using an Embedding Triangle Eddy Current Sensing Film. *Sensors* **2021**, *21*, 2567. https://doi.org/10.3390/s21072567.
